# *De Novo* Assembly and Phasing of Dikaryotic Genomes from Two Isolates of *Puccinia coronata* f. sp. *avenae*, the Causal Agent of Oat Crown Rust

**DOI:** 10.1128/mBio.01650-17

**Published:** 2018-02-20

**Authors:** Marisa E. Miller, Ying Zhang, Vahid Omidvar, Jana Sperschneider, Benjamin Schwessinger, Castle Raley, Jonathan M. Palmer, Diana Garnica, Narayana Upadhyaya, John Rathjen, Jennifer M. Taylor, Robert F. Park, Peter N. Dodds, Cory D. Hirsch, Shahryar F. Kianian, Melania Figueroa

**Affiliations:** a Department of Plant Pathology, University of Minnesota, St. Paul, Minnesota, USA; b Supercomputing Institute for Advanced Computational Research, University of Minnesota, Minneapolis, Minnesota, USA; c Centre for Environment and Life Sciences, Commonwealth Scientific and Industrial Research Organization, Agriculture and Food, Perth, WA, Australia; d Research School of Biology, Australian National University, Canberra, ACT, Australia; e Leidos Biomedical Research, Frederick, Maryland, USA; f Center for Forest Mycology Research, Northern Research Station, USDA Forest Service, Madison, Wisconsin, USA; g Agriculture and Food, Commonwealth Scientific and Industrial Research Organization, Canberra, ACT, Australia; h Plant Breeding Institute, Faculty of Agriculture and Environment, School of Life and Environmental Sciences, University of Sydney, Narellan, NSW, Australia; i USDA-ARS Cereal Disease Laboratory, St. Paul, Minnesota, USA; j Stakman-Borlaug Center for Sustainable Plant Health, University of Minnesota, St. Paul, Minnesota, USA; University of Córdoba

**Keywords:** effectors, genomics, oat, rust fungi, virulence

## Abstract

Oat crown rust, caused by the fungus *Pucinnia coronata* f. sp. *avenae*, is a devastating disease that impacts worldwide oat production. For much of its life cycle, *P. coronata* f. sp. *avenae* is dikaryotic, with two separate haploid nuclei that may vary in virulence genotype, highlighting the importance of understanding haplotype diversity in this species. We generated highly contiguous *de novo* genome assemblies of two *P. coronata* f. sp. *avenae* isolates, 12SD80 and 12NC29, from long-read sequences. In total, we assembled 603 primary contigs for 12SD80, for a total assembly length of 99.16 Mbp, and 777 primary contigs for 12NC29, for a total length of 105.25 Mbp; approximately 52% of each genome was assembled into alternate haplotypes. This revealed structural variation between haplotypes in each isolate equivalent to more than 2% of the genome size, in addition to about 260,000 and 380,000 heterozygous single-nucleotide polymorphisms in 12SD80 and 12NC29, respectively. Transcript-based annotation identified 26,796 and 28,801 coding sequences for isolates 12SD80 and 12NC29, respectively, including about 7,000 allele pairs in haplotype-phased regions. Furthermore, expression profiling revealed clusters of coexpressed secreted effector candidates, and the majority of orthologous effectors between isolates showed conservation of expression patterns. However, a small subset of orthologs showed divergence in expression, which may contribute to differences in virulence between 12SD80 and 12NC29. This study provides the first haplotype-phased reference genome for a dikaryotic rust fungus as a foundation for future studies into virulence mechanisms in *P. coronata* f. sp. *avenae*.

## IMPORTANCE

Disease management strategies for oat crown rust are challenged by the rapid evolution of *Puccinia coronata* f. sp. *avenae*, which renders resistance genes in oat varieties ineffective. Despite the economic importance of understanding *P. coronata* f. sp. *avenae*, resources to study the molecular mechanisms underpinning pathogenicity and the emergence of new virulence traits are lacking. Such limitations are partly due to the obligate biotrophic lifestyle of *P. coronata* f. sp. *avenae* as well as the dikaryotic nature of the genome, features that are also shared with other important rust pathogens. This study reports the first release of a haplotype-phased genome assembly for a dikaryotic fungal species and demonstrates the amenability of using emerging technologies to investigate genetic diversity in populations of *P. coronata* f. sp. *avenae*.

## INTRODUCTION

Cultivated oat (*Avena sativa*) ranks sixth in global production among cereals like maize, rice, and wheat (1). In recent years, the demonstrated health benefits of oats and its expanded commercial applications have increased demand for the crop (2). Crown rust, caused by the pathogenic fungus *Puccinia coronata* f. sp. *avenae*, is the most devastating disease affecting production in nearly every oat-growing region worldwide (2, 3), with yield losses due to infection reaching 50% (4).

*Puccinia coronata* f. sp. *avenae* is a macrocyclic and heteroecious rust fungus (*Puccinales*, *Basidiomycota*) (2). Asexual or clonal reproduction of *P. coronata* f. sp. *avenae* occurs in oat and in its wild relatives and involves repeated infection cycles mediated by urediniospores, which can perpetuate infection indefinitely (2). The infection process involves germination of urediniospores on the leaf surface, appressorium and penetration peg differentiation to allow host entry through a stomate, formation of a substomatal vesicle, the establishment of a colony by hyphal proliferation, and finally sporulation to produce more urediniospores. During infection, the fungus also forms haustoria, specialized feeding structures that allow nutrient uptake and secretion of effector proteins into the host cells (5). During the asexual cycle, *P. coronata* f. sp. *avenae* is dikaryotic, with each urediniospore containing two haploid nuclei, while the sexual cycle involves meiosis and infection of an alternate host of the genus *Rhamnus* (e.g., common buckthorn) by haploid spores and subsequent gamete fusion to reestablish the dikaryotic stage (2). Thus, the sexual cycle contributes to oat crown rust outbreaks both by generating an additional source of inoculum and by reassorting genetic variation in the pathogen population.

Disease management strategies for oat crown rust rely heavily on breeding for race-specific resistance (2). However, *P. coronata* f. sp. *avenae* rapidly evolves virulence to new resistance genes, and field populations are highly polymorphic, with high numbers of races (pathotypes), which limits the efficacy of this approach (6). Resistance to *P. coronata* f. sp. *avenae* in *Avena* spp. conforms to the classical gene-for-gene model (7, 8) and is conditioned by dominant resistance (*R*) genes, which mediate recognition of cognate avirulence (*Avr*) factors in the pathogen. Plant *R* genes typically encode intracellular nucleotide binding and leucine-rich repeat (NLR) receptor proteins, which detect specific pathogen effector proteins and induce a localized hypersensitive response (9, 10). Evolution of new virulence traits occurs due to changes in effector genes that allow the pathogen to escape recognition (11). Several *Avr* genes identified in the model flax rust *Melampsora lini* encode secreted proteins expressed in haustoria that are recognized inside host cells (12, 13). However, no *Avr* genes have been identified in *P. coronata* f. sp. *avenae*, and the biological mechanisms generating genetic variability in *P. coronata* f. sp. *avenae* are unknown. Since *P. coronata* f. sp. *avenae* is dikaryotic, a virulence phenotype requires the loss of the avirulence function of both alleles at the effector locus, and thus the emergence of virulent strains can be enhanced by sexual recombination. Nevertheless, the high diversity of virulence phenotypes in asexual populations suggests that additional molecular mechanisms, like high mutational rates, somatic hybridization, and somatic recombination, play roles in generating variability in *P. coronata* f. sp. *avenae* (14–16).

Given their biotrophic lifestyle, most rust fungi are recalcitrant to *in vitro* culturing and genetic transformation, which hinders molecular studies of pathogenicity. Nevertheless, genome sequencing of a few rust species has provided insights into the biology and adaptations associated with parasitic growth (17–24). These resources have enabled the prediction of effector candidates and, in some instances, identification of *Avr* genes (13, 25). However, the large genome sizes of rust fungi sequenced to date (90 to 200 Mbp) compared to those of other pathogenic fungi (26–29) and the high repetitive DNA content (over 50%) hamper *de novo* genome assembly from short-read sequencing, which leads to high fragmentation, misassembly errors, and merging of two distinct haplotype sequences. Moreover, to the best of our knowledge, currently available rust genome assemblies represent collapsed mosaics of sequences derived from both haplotypes in the dikaryon and do not account for structural variation between haplotypes. Single-molecule real-time (SMRT) sequencing has emerged as a powerful technology to achieve high-contiguity assembly of even repeat-rich genomes (30), and recently released algorithms enable the resolution of haplotypes in diploid genomes (31).

Here, we document the assembly of draft genome sequences for two *P. coronata* f. sp. *avenae* isolates with contrasting virulence phenotypes by using SMRT sequencing and the FALCON assembler and FALCON-Unzip for haplotype resolution (31). The contiguity of the *P. coronata* f. sp. *avenae* assemblies is greatly improved compared to that of previous short-read *de novo* assemblies of rust species (20–22). We separately assembled the two haplotypes for over 50% of the haploid genome of each isolate. This revealed many structural differences between haplotypes and isolates, including large insertions/deletions (indels) covering both intergenic and coding regions. The *P. coronata* f. sp. *avenae* genomes were annotated by utilizing expression data from different tissue types and life stages, and a catalog of predicted secreted effectors was generated. To our knowledge, this study and that of Schwessinger and colleagues on the wheat stripe rust pathogen *Puccinia striiformis* f. sp. *tritici* (32) provides the first report of genome-wide haplotype resolution of dikaryotic rust fungi and the foundation to investigate the evolution of virulence factors and the contribution of haplotype variation to the pathogenicity of *P. coronata* f. sp. *avenae*.

## RESULTS AND DISCUSSION

### *Puccinia coronata* f. sp. *avenae* isolates 12SD80 and 12NC29 show distinct virulence profiles.

**FIG 1:**
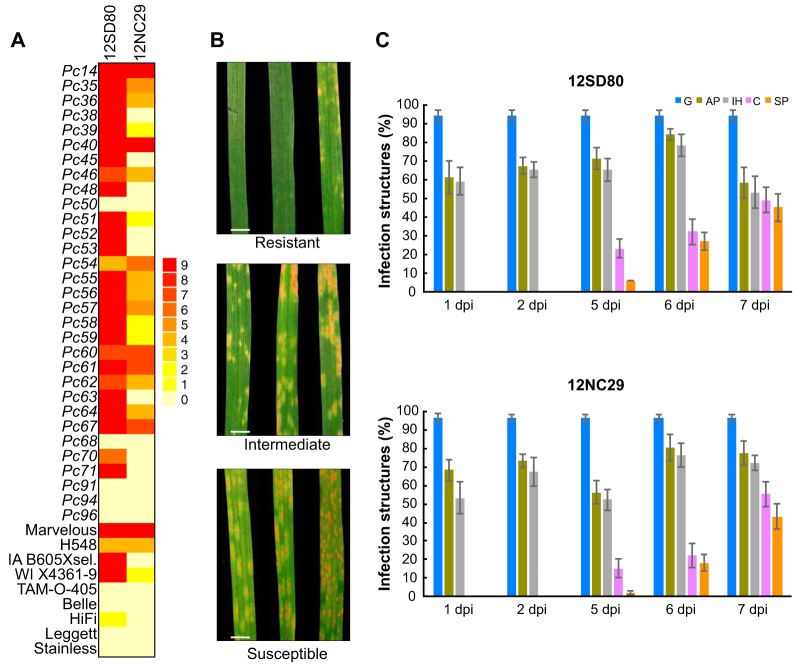
Phenotypic variation of *P. coronata* f. sp. *avenae* isolate virulence and colonization patterns in susceptible oat. (A) Heatmap showing virulence profiles of 12SD80 and 12NC29 on a set of 40 oat differential lines. Infection type scores were converted to a 0-to-9 numeric scale for heatmap generation. (B) Photographs represent examples of infection types corresponding to full resistance (with a score of 0 to 4) or intermediate resistance (5 or 6), as well as susceptibility (7 to 9). Example photographs show infection of 12SD80 on representative lines of the standard U.S. oat differential set. Scale bar = 0.5 cm. (C) Quantification of infection structures of *P. coronata* f. sp. *avenae* isolates in the susceptible oat line Marvelous at 1, 2, 5, 6, and 7 dpi. Graphs show the percentages of urediniospores that have germinated (G) and the percentages of germinated spores that formed appressoria (AP), substomatal vesicles or primary infection hyphae (IH), established colonies (C), and sporulating colonies (SP). Error bars represent standard errors of results from three independent replicates.

To build comprehensive genomic resources for virulence studies in *P. coronata* f. sp. *avenae*, we selected from the 2012 USDA-ARS annual rust survey two isolates, 12NC29 and 12SD80, that show contrasting virulence profiles on an oat differential set (Fig. 1A and B). Isolate 12SD80 is virulent on a broader range of oat differentials than isolate 12NC29, although recently released *P. coronata* resistance genes (*Pc91*, *Pc94*, *Pc96*) are effective against both isolates. Despite the different virulence profiles on specific *P. coronata* resistance (Pc) genes, both isolates showed similar levels of infection progression over a 7-day time course on the susceptible oat variety Marvelous (Fig. 1C). More than 90% of urediniospores germinated, and of them, more than 60% differentiated an appressorium (penetration structure) in the first 24 h of infection. Established colonies and the first signs of sporulation were detected by 5 days postinfection (dpi), and 40 to 50% of infection sites displayed sporulation by 7 dpi. Thus, both *P. coronata* f. sp. *avenae* isolates were equally pathogenic in the absence of effective *P. coronata* resistance genes.

### *De novo* genome assembly and haplotype-phasing of *P. coronata* f. sp. *avenae* isolates.

High-molecular-weight DNA (>50 kbp) was extracted from germinated urediniospores of 12SD80 and 12NC29, and long-read sequence data were generated using SMRT sequencing. This yielded approximately 20.9 and 25.9 Gbp of filtered subreads for 12SD80 and 12NC29, respectively. The mean and *N*_50_ subread lengths were 6,389 and 8,445 bp, respectively, for 12SD80, and 6,481 and 8,609 bp for 12NC29 (see Table S1 in the supplemental material), and subread distributions for both isolates extended to approximately 30,000 bp. Illumina sequencing was performed on the same samples and yielded approximately 6 and 7 Gbp of sequence information for 12SD80 and 12NC29, respectively.

10.1128/mBio.01650-17.7 TABLE S1  Summary statistics for SMRT sequencing reads. Download TABLE S1, DOCX file, 0.1 MB. Copyright © 2018 Miller et al. 2018 Miller et al. This content is distributed under the terms of the Creative Commons Attribution 4.0 International license.

**FIG 2:**
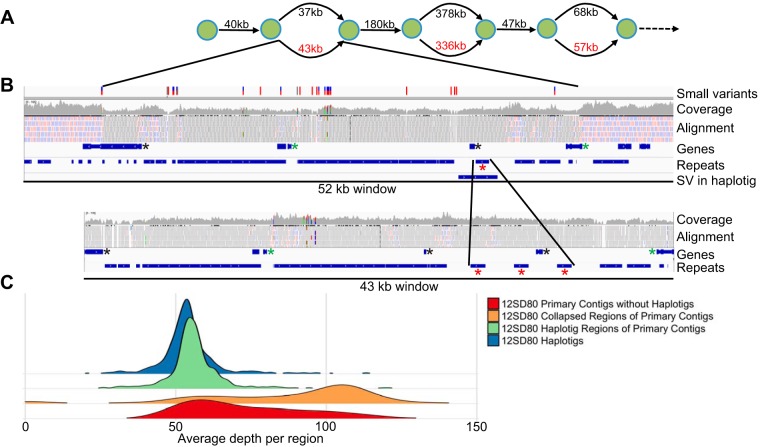
Characteristics of haplotig regions in a primary contig for the *P. coronata* f. sp. *avenae* isolate 12SD80. (A) Schematic depicting the first three haplotig regions of the largest primary contig in 12SD80 (000000f). The green circles represent nodes in the assembly graph, and the numbers represent the distance between nodes for the primary contig (upper path, black) and haplotigs (lower path, red). (B) IGV genome browser view of the first haplotig-associated region of 12SD80 contig 000000f (upper panel) and the corresponding haplotig (lower panel). The top track shows SNPs and indels between haplotypes. The next track shows the coverage of short reads mapping to the assembly, and below that is the raw alignment evidence. Uniquely mapping reads are shown in red (negative strand orientation) and blue (positive strand orientation), while gray indicates reads mapping to multiple locations. Annotated genes and repeats are shown in separate tracks, and the bottom track for the primary contig shows structural variations (SVs). Red asterisks indicate a repeat element that has undergone a tandem expansion in the haplotig, green asterisks indicate properly phased allele pairs, and black asterisks indicate genes not assigned as an allele, likely due to fragmentation or substantial sequence divergence. (C) Density histograms of the mean coverage depth of collapsed and haplotig regions of primary contigs, haplotigs, and primary contigs without haplotigs in 12SD80.

Given that *P. coronata* f. sp. *avenae* urediniospores are dikaryotic, the diploid aware assembler FALCON in combination with FALCON-Unzip (31) was used to first assemble the genomes of 12NC29 and 12SD80 and then distinguish regions of homology and divergence between haplotypes. Initial assembly with FALCON produces a set of primary contigs and a set of alternate contigs that represent regions of divergence between haplotypes and are associated with a homologous region on a primary contig. FALCON-Unzip uses the contigs from FALCON as the input, and heterozygous single-nucleotide polymorphisms (SNPs) and structural variants are used to generate final primary contigs and haplotigs. It is important to note that the assembly is a representation of haplotype blocks and that collapsed regions of primary contigs can contain sequences from both haplotypes. Because of this, the primary contigs should contain the equivalent of one haploid genome, and haplotigs represent the total sequence placed in alternate assembly paths relative to those of each individual primary contig (Fig. 2A). Genome assembly of 12SD80 resulted in 603 primary contigs with a total size of 99.2 Mbp and a contig *N*_50_ of 268.3 kbp, while 777 primary contigs with a total size of 105.2 Mbp and a contig *N*_50_ of 217.3 kbp were assembled for 12NC29 (Table 1). These assemblies demonstrate the advantage of long-read assembly to improve contiguity compared to that of previous short-read assemblies of other rust species. For example, the genome assembly of the wheat stripe rust fungus *Puccinia striiformis* f. sp. *tritici* contained more than 29,000 contigs, with an *N*_50_ of 5.1 kbp (19), and the assembly of the flax rust fungus, *Melampsora lini*, has 21,000 scaffolds, with an *N*_50_ of 31 kbp (22). The contiguity of our *P. coronata* f. sp. *avenae* genome assemblies are comparable to the scaffolding efficiency of the large-insert Sanger sequence-based assemblies of the poplar rust fungus *Melampsora larici-populina* and the wheat stem rust fungus *Puccinia graminis* f. sp. *tritici*, which contained 462 and 392 scaffolds, respectively (17). However, the *Melampsora larici-populina* and *P. graminis* f. sp. *tritici* scaffolds contain approximately 3.5 and 7 Mbp of missing data, respectively, as gaps between contigs. The estimated genome sizes of 12SD80 and 12NC29 are in the range of those of other related rusts, such as *P. graminis* f. sp. *tritici* (92 Mbp) (17, 18) and *P. striiformis* f. sp. *tritici* (65 to 130 Mbp) (19, 21, 24), and in agreement with nuclear DNA fluorescence intensity measurements of haploid pycniospores, suggesting about a 15% larger genome size of *P. coronata* f. sp. *avenae* than that of *P. graminis* f. sp. *tritici* (33). Similarly, a preliminary genome assembly of an Australian isolate of *P. coronata* f. sp. *avenae* based on Illumina short reads suggested a genome size of 110 Mbp (R. F. Park, P. Zhang, and C. M. Dong, unpublished data). On the other hand, Tavares et al. (34) reported a haploid genome size of approximately 244 Mbp based on nuclear fluorescence for a *P. coronata* isolate obtained from *Avena sterilis*. Given the broad host range of *P. coronata* (2), this isolate may represent a different *forma specialis*.

**TABLE 1:** Assembly metrics and evaluation

Parameter	Value for:			
	12SD80 primary contigs	12SD80 haplotigs	12NC29 primary contigs	12NC29 haplotigs
No. of contigs ≥0 bp	603	1,033	777	950
No. of contigs ≥50,000 bp	475	372	560	403
Total length (Mb)	99.2	51.3	105.2	61.0
Total length of contigs ≥50,000 bp (Mb)	94.9	36.2	98.0	49.4
Size of largest contig (Mb)	1.39	0.35	1.19	0.48
GC content (%)	44.7	44.9	44.7	44.9
*N*_50_ (kb)	268.3	77.8	217.3	121.2
% of complete BUSCOs	90.4	57.9	89.6	72.1
% of complete and single-copy BUSCOs	85.9	57.2	84.1	69.7
% of complete and duplicated BUSCOs	4.5	0.7	5.5	2.4
% of fragmented BUSCOs	3.1	3.8	2.8	4.1
% of missing BUSCOs	6.5	38.3	7.6	23.8

Totals of 1,033 and 950 haplotigs were assembled for 12SD80 and 12NC29, respectively, comprising 52% of the haploid genome size in each case (Table 1). Haplotig sequences were aligned to primary contigs to identify corresponding regions as well as unphased sequences. This is illustrated for the largest primary contig in 12SD80 in Fig. 2A. Numerous small variants were detected in the first haplotig-associated region in this primary contig and the corresponding haplotig by alignment of Illumina DNA reads to primary contigs and haplotigs simultaneously (Fig. 2B). The haplotig also contains a tandem repeat expansion relative to the sequence of the primary contig, while the flanking collapsed regions in the primary contig are less varied. The variation in this region likely explains why an alternate path in the assembly graph led to the phasing of this genomic region. The Illumina read depth (coverage) in the haplotig region is lower than that of the flanking collapsed regions, as is expected considering that haplotig-associated regions represent a single haplotype, whereas most collapsed regions in primary contigs represent both haplotypes. In addition, reads in the collapsed region map uniquely in the genome, while those in the haplotig region map to multiple sites.

To validate haplotype phasing more extensively, we calculated genome-wide coverage for haplotig-associated regions and regions without haplotigs (referred to as collapsed) within primary contigs, as well as haplotigs. Haplotigs and haplotig regions of primary contigs in 12SD80 showed tight coverage distribution, with mean coverages of 56.3 and 58.7, respectively, while collapsed regions had a mean coverage of 103.6 but showed a broader distribution (Fig. 2C). Similarly, 12NC29’s mean coverages of haplotigs, phased regions, and collapsed regions of primary contigs were 62.6, 64.3, and 91.0, respectively (Fig. S1A). Some collapsed regions of primary contigs show lower coverage than others. These primary contigs may represent genomic locations with high levels of divergence between the haplotypes and complex rearrangements or very large insertions/deletions (indels) that prevent proper assignment of the corresponding haplotype sequence to a haplotig, as discussed by Chin et al. (31). This might result in the presence of haplotype-specific sequences in primary contigs. Additionally, some primary contigs did not contain any associated haplotigs, which might also be due to high levels of haplotype divergence leading to the assembly of haplotypes as two separate primary contigs. Consistently with this, primary contigs without haplotigs showed a lower coverage distribution than those with associated haplotigs (Fig. 2C). The contigs present in the lower tail of the distribution are likely separately assembled haplotype sequences, whereas the contigs in the upper tail of the distribution for this class have two haplotypes that are collapsed. In 12SD80 and 12NC29, there were 176 and 312 primary contigs without haplotigs, respectively, totaling 11.1 and 17.5 Mbp. If these do represent separately assembled haplotypes, then this may partly explain the approximately 6-Mbp-larger primary contig assembly size for 12NC29. To determine how many primary contigs without haplotigs might be separately assembled haplotypes, we calculated the percentage of primary contigs without haplotigs that align to other primary contigs in the assembly. The longest alignments between each query (primary contigs without haplotigs) and reference (all primary contigs) were extracted, and we considered only those instances in which the alignment size between the reference and query was greater than half of the query length. Using these criteria, 33.1% and 33.4% of primary contigs without haplotigs in 12SD80 and 12NC29, respectively, align to another primary contig. This suggests that many of the primary contigs without haplotigs are likely separately assembled haplotype sequences but that due to high divergence were not associated during FALCON-Unzip assembly. We also compared repeat and coding region abundance on primary contigs without haplotigs and phased portions of primary contigs (regions with associated haplotigs) to investigate whether repeat content could have impacted the phasing efficiency of the assembler. Phased portions of primary contigs contained 30.4% and 29.2% coding regions in 12SD80 and 12NC29, respectively, and 53.3% and 53.7% repeat regions. In contrast, primary contigs without haplotigs contained 20.3% and 19.9% coding regions in 12SD80 and 12NC29, respectively, and 64.5% and 68.3% repeat regions, indicating lower gene density and a higher repeat abundance on primary contigs without haplotigs. Higher repeat percentages on these contigs likely contributed to difficulties in phasing these regions. Similar results were found for the stripe rust fungus (32). The ability to phase the genome assembly into primary contigs and haplotigs in this fashion represents a significant advance for comparing haplotype compositions in dikaryotic fungi.

10.1128/mBio.01650-17.1 FIG S1  Coverage of 12NC29, GC content of genome assemblies, and inter-isolate read mapping coverage of isolate singleton and orthologous genes. (A) Density histograms of mean coverage depth of collapsed and haplotig regions of primary contigs, haplotigs, and primary contigs without haplotigs in 12NC29. (B) GC content distribution of contigs from 12NC29 and 12SD80 assemblies. (C) Reads from one isolate were mapped to the other isolate to assess coverage of isolate singleton and orthologous genes on primary contigs. Density histograms of average coverage depth per gene for 12SD80 (left) and 12NC29 (right) are shown. Download FIG S1, PDF file, 2.5 MB. Copyright © 2018 Miller et al. 2018 Miller et al. This content is distributed under the terms of the Creative Commons Attribution 4.0 International license.

### Assessment of genome completeness and repetitive DNA content.

To assess the completeness of the *P. coronata* f. sp. *avenae* genome assemblies, highly conserved fungal genes were mapped in the primary contigs and haplotigs with BUSCO (35). Approximately 90% of the BUSCO genes were present as complete sequences, and nearly an additional 3% were present as fragmented copies in the primary contigs of both genome assemblies (Table 1). One additional BUSCO gene not present in the primary contigs was found on a haplotig in 12SD80, while no unique BUSCO genes were found in 12NC29 haplotigs. Fourteen out of the 290 possible BUSCO genes (4.8%) were missing in both isolates, which suggests the presence of regions in the *P. coronata* f. sp. *avenae* genome that are difficult to assemble. A search for telomere repeat sequences at the ends of all contigs detected 11 unique telomeres in 12NC29 and 15 in 12SD80, out of an estimated 16 to 20 chromosomes (36). Overall, these results indicated that the primary contigs are a good representation of the core dikaryotic genome of *P. coronata* f. sp. *avenae*.

RepeatMasker detected interspersed repeats covering about 53% of the assembled *P. coronata* f. sp. *avenae* genomes (primary contigs and haplotigs combined) (Table 2), similar to what occurs with other rust fungi, which are typically in the range of 35 to 50% (17, 21, 22). The most prevalent repetitive elements belonged to the long terminal repeat (LTR) retroelement class (20% of the genome), which was also found to be the most abundant class in *P. graminis* f. sp. *tritici* and *Melampsora larici-populina* (17, 24), while DNA elements accounted for about 15% of the genome. The GC content was approximately 45% for primary contigs and haplotigs in both *P. coronata* f. sp. *avenae* isolates (Table 1), which is consistent with those of other rust species, such as *Melampsora lini* (41%) (22). The distribution of GC contents in individual contigs (Fig. S1B) did not display a bimodal distribution indicative of the presence of AT-rich regions, such as those observed in fungi that use repeat-induced point mutation (RIP) to inhibit transposon proliferation (37).

**TABLE 2:** Proportions of repeated sequence content in *P. coronata* f. sp. *avenae* isolates

Repeat class	% for:	
	12SD80	12NC29
Total	52.76	53.66
SINEs	0.02	0.01
LINEs	0.84	0.95
LTR elements	20.10	20.18
DNA elements	14.50	15.56
Unclassified	16.02	16.24
Small RNA	0.05	0
Satellites	0.12	0.05
Simple repeats	1.58	1.22
Low complexity	0.11	0.12

### Gene annotation and orthology prediction reveals phased allele pairs within isolates and orthologs between isolates.

For each *P. coronata* f. sp. *avenae* isolate, transcriptome sequencing (RNA-seq) reads from germinated spores, isolated haustoria, and infected oat leaves at 2 and 5 dpi (Table S2) were pooled and used to generate both *de novo* and genome-guided transcriptome assemblies using Trinity v2.4.0 (38). These assemblies were used as transcriptional evidence in the Funannotate pipeline, along with alignment evidence from publicly available expressed sequence tag (EST) clusters for *Pucciniamycotina* species. We annotated, in total, 17,248 and 17,865 genes on primary contigs for 12SD80 and 12NC29, respectively (Table 3), which is similar to the haploid gene content of other rust fungal genomes (17, 22). An additional 9,548 and 10,936 genes were annotated on haplotigs for 12SD80 and 12NC29, respectively.

10.1128/mBio.01650-17.8 TABLE S2  Alignment statistics of RNA-seq reads mapping to *P. coronata* f. sp. *avenae* assemblies (primary contigs). GS, 2, 5, and H indicate germinated spores, 2-dpi samples, 5-dpi samples, and haustoria samples, respectively. R1, R2, and R3 designate the different biological replicates. Download TABLE S2, DOCX file, 0.02 MB. Copyright © 2018 Miller et al. 2018 Miller et al. This content is distributed under the terms of the Creative Commons Attribution 4.0 International license.

**TABLE 3:** Gene, allele, and ortholog content in *P. coronata* f. sp. *avenae* genome assemblies

Parameter^a^	Value for:	
	12SD80	12NC29
Total no. of genes (P and H)	26,796	28,801
Mean gene length for all genes (bp)	1,516	1,518
% of genome covered by genes	27.0	26.3
Total no. of genes on P	17,248	17,865
Total no. of genes on H	9,548	10,936
No. of allele pairs on P and H	6,664	7,789
No. of haplotype singleton genes on P	2,162	2,311
No. of haplotype singleton genes on H	2,033	2,154
No. of effectors on P	529	549
No. of effectors on H	320	351
No. of effectors on P in allele pairs	268	277
No. of effectors on H in allele pairs	262	276
No. of haplotype singleton effectors on P	42	61
No. of haplotype singleton effectors on H	49	62
No. of orthologous effectors on P between isolates	336	327
No. of isolate singleton effectors on P	184	216

a P and H, primary contigs and haplotigs, respectively.

To identify putative allele pairs in the phased assemblies, we searched for genes on primary contigs that had an ortholog present on the corresponding haplotig using Proteinortho (39) in synteny mode to account for gene order (Table 3). A total of 6,664 and 7,789 such allele pairs were identified in 12SD80 and 12NC29, respectively. About 2,000 haplotype singletons with no orthologs in a corresponding region were also detected in haplotig regions of primary contigs with a similar number in haplotigs (Table 3). These singletons may represent haplotype-specific genes with presence/absence variation or genes with substantial sequence variation, which prevents orthology detection. Alternatively, some singletons may have an allele present on a corresponding haplotig but, due to the higher fragmentation of haplotigs, such an allele could not be detected (see the asterisks in Fig. 2 for an example). A total of 38.8% and 39.4% of singletons on primary contigs in 12SD80 and 12NC29, respectively, had a full-length BLAST hit (E value < 0.00001) of sequence similarity of between 69 and 100% on a corresponding haplotig, indicating the presence of an allelic sequence. Differences in annotation between the primary contigs and haplotigs, for instance due to polymorphisms that disrupt their coding potential, may explain why these were not called orthologs. In 12SD80, in total, 97 and 24 singletons in primary contigs and haplotigs, respectively, had no significant BLAST hit. These results were similar to those for 12NC29, as 113 and 39 singletons in primary contig and haplotigs, respectively, had no significant BLAST hit. Overall, this analysis supports the high haplotype divergence in both isolates.

We also examined gene orthology between isolates and identified 9,764 orthologous groups (~55% of all genes) containing either (i) two orthologous genes, one from each isolate with no allele pairs, (ii) an allele pair from one isolate with an unpaired gene from the other, or (iii) two allele pairs, one from each isolate. Isolate singletons might represent presence/absence polymorphisms or might be due to sequence divergence or genome rearrangements preventing orthology detection. Therefore, we examined gene coverage by cross-mapping Illumina reads from each isolate onto the other assembly (Fig. S1C). The isolate singleton genes in 12SD80 and 12NC29 included 558 and 1,174 genes, respectively, with low coverage (<30×), suggesting that they represent the presence/absence polymorphisms, while the remainder showed higher coverage (30× to 200×), indicating that homologs may be present in the two isolates. Taken together, these findings indicate a high level of gene content variation between haplotypes and isolates of *P. coronata* f. sp. *avenae*. Sequencing a larger sample of *P. coronata* f. sp. *avenae* isolates will help determine the number of conserved (core) genes versus isolate-specific genes in this species.

### Functional annotation of *P. coronata* f. sp. *avenae* genomes.

**FIG 3:**
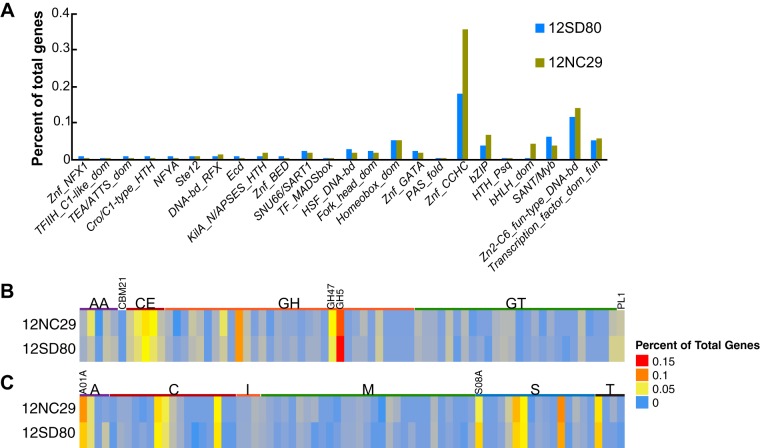
Functional annotation of transcription factors, CAZymes, and Merops proteases in *P. coronata* f. sp. *avenae* isolates. (A) Percentages of genes predicted to encode members of various fungal transcription factor classes based on InterProScan annotation. (B) Heatmap showing numbers of genes annotated as members of CAZyme families in the following classes: auxiliary activities (AA), carbohydrate-binding modules (CBM), carbohydrate esterases (CE), glycoside hydrolases (GH), glycosyltransferases (GTs), and polysaccharide lyases (PL). Expanded families GH5 and GH47 are indicated. (C) Heatmap showing the numbers of genes annotated as members of the Merops families of aspartic acid (A), cysteine (C), metallo protease (M), serine protease (S), and threonine protease (T) or peptidase inhibitors (I). Expanded families A01A and S08A are indicated.

Gene ontology (GO) term abundances of annotated genes on primary contigs and haplotigs combined were very similar between isolates, with no significant GO term enrichments or depletions. Examination of KEGG pathway annotations (40) indicated that, as observed for other rust fungi (17, 22, 24), the *P. coronata* f. sp. *avenae* genomes lacked nitrate and nitrite assimilation genes. The assemblies did contain the enzymes glutamine synthetase (K01915), glutamate synthase (K00264), and glutamate dehydrogenase (K00260), which are putatively involved in nitrogen assimilation from host-derived amino acids. Enzymes of the sulfate assimilation pathway were also absent in the two *P. coronata* f. sp. *avenae* isolates. Notably, sulfite reductase was missing from both assemblies, as was observed for *P. graminis* f. sp. *tritici* (17). These observations are consistent with the loss of nitrate, nitrite, and sulfate assimilation pathways associated with the evolution of obligate biotrophy in rust fungi (17, 22). Most categories of transcription factor (TF) families showed low abundance in both isolates except the CCHC zinc finger class (IPR001878), which has 103 members in 12NC29 and 48 in 12SD80 (Fig. 3A). This family was also expanded in *P. graminis* f. sp. *tritici* and *Melampsora larici-populina* relative to in other fungi (17) and are of particular interest, as zinc finger TFs are hypothesized to play roles in effector regulation (41).

### Heterozygosity in the dikaryotic genome of *P. coronata* f. sp. *avenae*.

Heterozygous small variants, including SNPs, indels, and multiple-nucleotide polymorphisms (MNPs), were identified by mapping Illumina reads to only primary contigs in each isolate. We detected 3.45 and 4.60 heterozygous variants/kbp (including 2.68 and 3.62 SNPs/kbp) in 12SD80 and 12NC29, respectively. These heterozygosity rates are in line with genome-wide estimates of 1 to 15 heterozygous SNPs/kbp for other *Puccinia* spp. (18, 19, 21, 24), although such estimates may be influenced by differences in variant calling methods and parameters, residual assembly errors, read length, and coverage and may differ between isolates of a species. When Illumina reads from 12SD80 were mapped to the 12NC29 primary contig reference, we detected a total of 3.48 heterozygous and 2.31 homozygous variants/kbp. In the reciprocal comparison, 5.60 heterozygous and 1.75 homozygous variants/kbp were identified, indicating substantial variation between isolates as well as between haplotypes.

The majority of variants between haplotypes were found in intergenic regions (Fig. S2A), and these occurred at a higher frequency (3.66 and 4.88 variants/kbp in 12SD80 and 12NC29, respectively) than that of variants in genic regions (2.86 and 3.76 variants/genic kbp). Heterozygosity rates were higher in the haplotig regions of primary contigs (4.36 and 5.50 variants/kbp in 12SD80 and 12NC29, respectively) than in collapsed regions (1.06 and 1.27 variants/kbp). Genes in allele pairs present in phased regions of primary contigs contain more variants (3.31 and 4.03 variants/kbp in 12SD80 and 12NC29, respectively) than those in collapsed regions (2.01 and 2.87 variants/kbp in 12SD80 and 12NC29, respectively). Similarly, numbers of synonymous substitutions per kilobase pair in allele pairs of haplotig regions (0.90 and 1.07 in 12SD80 and 12NC29, respectively) were elevated compared to genes in collapsed regions (0.49 and 0.68 in 12SD80 and 12NC29, respectively). These observations are consistent with haplotigs containing regions of divergence between haplotypes. We did not detect any enrichment of GO categories in any of the sets of collapsed genes, allelic genes, or singletons on primary contigs.

10.1128/mBio.01650-17.2 FIG S2  Small sequence variants and structural variation between haplotypes of 12SD80 and 12NC29. (A) Genome-wide characterization of SNPs and small indels classified by genomic location as intergenic (dark green), 1 kbp downstream (orange) or upstream (purple) of a gene, and exonic (red) and intronic (light green) in 12SD80 and 12NC29. (B) Structural variation between haplotigs and primary contigs that overlap annotated genes. Colors indicate different classes of SV (shown in the key). Graphs in panels C and D show size distributions of structural variants from 50 to 10,000 bp identified using Assemblytics in haplotigs relative to primary contigs of 12SD80 and 12NC29, respectively. (E) Distribution of small variants in and around predicted effectors on primary contigs of 12SD80 and 12NC29. The key is the same as that shown in panel A. (F) SV types in predicted effector genes, as in panel B. Download FIG S2, EPS file, 1.5 MB. Copyright © 2018 Miller et al. 2018 Miller et al. This content is distributed under the terms of the Creative Commons Attribution 4.0 International license.

We also compared heterozygosity rates in *P. coronata* f. sp. *avenae* and the rust species *Melampsora larici-populina*, *Melampsora lini*, *P. striiformis* f. sp. *tritici*, and *Puccinia triticina* using a *k*-mer profile approach based on available Illumina reads with the software GenomeScope (42). In this analysis, homozygous genomes display a simple Poisson distribution in the *k*-mer profile plots, whereas heterozygous genomes give a bimodal profile. The *k*-mer profiles of most of these species (Fig. S3) showed bimodal profiles, which indicated fairly heterozygous genomes. This was less apparent for *P. striiformis* f. sp. *tritici* and *Melampsora lini*, which may be explained by the shorter read lengths and lower-coverage data sets for these species. Heterozygosity levels calculated in this analysis were similar for all species but lower than levels detected by SNP calling.

10.1128/mBio.01650-17.3 FIG S3  GenomeScope analysis of rust species. Comparison of 21 *k*-mer profiles of 12SD80, 12NC29, *Melampsora larici-populina*, *Puccinia striiformis*, *Puccinia triticina*, and *Melampsora lini*. Overall heterozygosity rate estimates are shown in each graph. Download FIG S3, PDF file, 2.1 MB. Copyright © 2018 Miller et al. 2018 Miller et al. This content is distributed under the terms of the Creative Commons Attribution 4.0 International license.

To assess structural variation (SV) between haplotypes, we compared haplotigs to their corresponding aligned regions in primary contigs using Assemblytics, which detects three types of SV: large insertions/deletions; tandem expansions/contractions, which involve tandemly repeated sequences; and repeat expansions/contractions in which homologous regions are separated by regions with no homology in each sequence (43). The distributions of these classes of SV are very similar between the two isolates (Fig. S2C and S2D), with insertions/deletions and repeat expansions/contractions being more prevalent than tandem expansions/contractions. Such SV of between 50 and 10,000 bp in size represented 2.7% of the primary contig genome size in 12NC29 and 2.1% in 12SD80 and impacted 646 and 951 coding regions on primary contigs in 12SD80 and 12NC29, respectively (Fig. S2B).

### Prediction of secretome and candidate effectors.

Pathogen effectors are secreted proteins that manipulate host cell processes to facilitate infection but can also be recognized by host resistance genes (44). Thus, differences in virulence profiles between 12NC29 and 12SD80 (Fig. 1A) likely result from variation in their effector repertoires. We predicted 1,532 and 1,548 secreted proteins on primary contigs of 12SD80 and 12NC29, respectively, corresponding to about 9% of all protein-coding genes. Similarly, 941 and 1,043 secreted proteins (including 773 and 856 in allele pairs) were predicted on haplotigs in 12SD80 and 12NC29, respectively. About 35% of all secreted proteins were predicted as effectors by the EffectorP machine learning tool for fungal effector prediction (45) (Table 4). No enriched GO terms were detected among the predicted effectors, and the vast majority had no homologs with known or predicted functions (Table S3), as is commonly observed for fungal effectors (46).

10.1128/mBio.01650-17.9 TABLE S3  Nonredundant GO terms present in predicted effectors on primary contigs. Download TABLE S3, DOCX file, 0.05 MB. Copyright © 2018 Miller et al. 2018 Miller et al. This content is distributed under the terms of the Creative Commons Attribution 4.0 International license.

**TABLE 4:** Features of proteins encoded by genes in different expression clusters of *P. coronata* f. sp. *avenae*^a^

Isolate and cluster	No. of proteins	% CAZymes	% EffectorP	% NLS (LOCALIZER)	% ApoplastP
12SD80					
1	251	18.7	37.1	20.7	23.1
2	78	6.7	29.5	19.2	43.6
3	55	6.7	27.3	12.7	61.8
4	**111**	**2.7**	**35.1**	**30.6**	**8.1**
5	**173**	**5.3**	**36.4**	**24.9**	**16.8**
6	197	36.0	20.3	18.8	30.5
7	198	24.0	42.9	16.7	48.0

12NC29					
1	239	17.1	36.0	23.8	25.1
2	93	14.5	33.3	30.1	30.1
** **3	**129**	**6.6**	**41.9**	**19.4**	**10.1**
4	71	7.9	23.9	9.9	53.5
5	166	19.7	24.1	22.9	28.3
6	**179**	**5.3**	**36.3**	**27.9**	**20.1**
7	86	7.9	45.3	10.5	52.3
8	124	13.2	29.0	18.5	37.1
9	60	7.9	26.7	8.3	55.0

a Boldface indicates haustorially expressed clusters.

**FIG 4:**
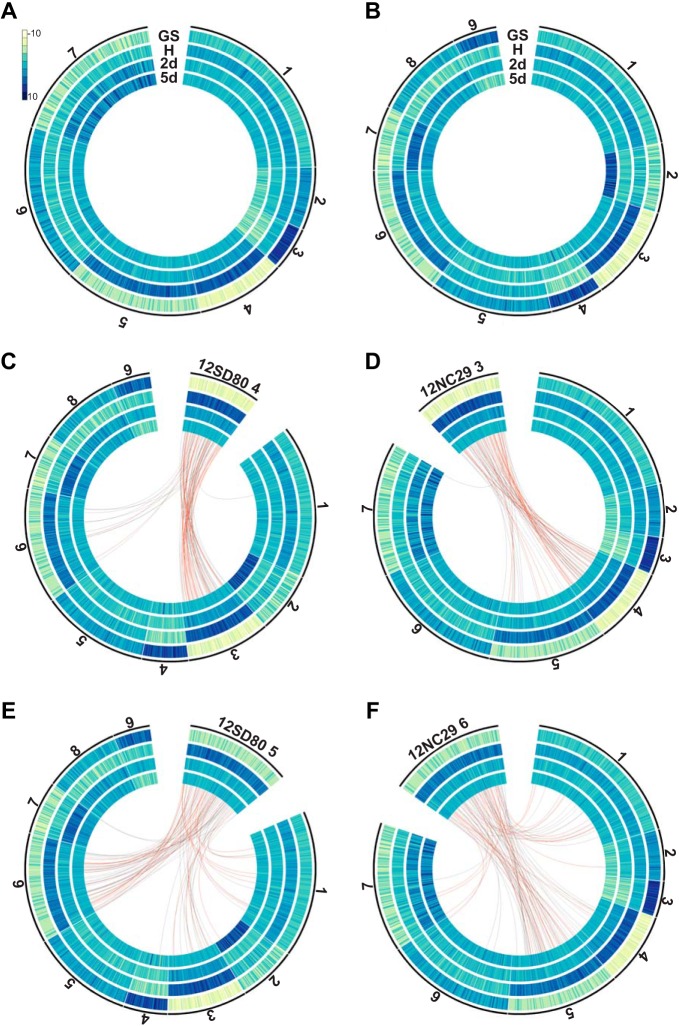
Clustering analysis of predicted secretome gene expression profiles and orthology in *P. coronata* f. sp. *avenae*. *k*-Means clustering of secretome genes of 12SD80 (A) and 12NC29 (B). Heatmaps show rlog-transformed expression values, with dark blue indicating high expression according to the scale. Cluster numbers are shown outside the graphs, and tracks show gene expression in germinated spores (GS), isolated haustoria (H), and infected tissues at 2 dpi (2d) and 5 dpi (5d). (C) Orthology relationships between genes in 12SD80 cluster 4 and all 12NC29 clusters are indicated by red (predicted effectors) and gray (other secreted proteins) lines. (D to F) Orthology relationships between genes in 12NC29 cluster 3 and all 12SD80 clusters (D), 12SD80 cluster 5 and all 12NC29 clusters (E), and 12NC29 cluster 6 and all 12SD80 clusters (F).

RNA-seq data sets from different tissue types were used to identify secreted protein genes in primary contigs of each isolate that were differentially expressed during infection, and similarly expressed genes were grouped using *k*-means clustering. This analysis detected seven distinct expression profile clusters for 12SD80 and nine for 12NC29 (Fig. 4A and B; Table 4). Genes in clusters 4 and 5 in 12SD80 showed high expression in haustorial samples and also relatively high expression in infected leaves, with those in cluster 4 showing the lowest expression in germinated urediniospores. Similar profiles were observed for clusters 3 and 6 in 12NC29. These expression patterns are consistent with those of previously identified secreted rust effectors that enter host cells, which show high expression in haustoria (5). About 35 to 40% of the secreted genes in these clusters were predicted as effectors by EffectorP (Table 4). These clusters also show relatively high proportions of genes encoding predicted nucleus-localized proteins and the lowest proportions of apoplast-localized proteins as predicted by ApoplastP (47) (Table 4), suggesting that these clusters are enriched for host-delivered effectors.

GO analysis detected an enrichment for molecular functions related to glycosyl hydrolase and peptidase activities in the *P. coronata* f. sp. *avenae* secretome (Fig. S4), which may indicate roles for these proteins during infection in the plant apoplast. Necrotrophic and hemibiotrophic plant-pathogenic fungi secrete large numbers of carbohydrate-active enzymes (CAZymes) including plant cell wall-degrading enzymes (PCWDEs) that are important for host invasion (48–50). However, biotrophs, such as rust fungi, contain far fewer of these enzymes, and their roles are less well defined, although roles in both plant cell wall degradation and fungal cell wall reorganization have been suggested based on expression data for *Melampsora larici-populina* and *P. graminis* f. sp. *tritici* (51). We detected 350 and 374 CAZymes in isolates 12SD80 and 12NC29, respectively, of which about 20% (75 and 76 CAZymes) were predicted to be secreted. This is consistent with estimates for other biotrophs from a fungal-kingdom-wide analysis of secreted proteins (52). Secreted CAZymes were most abundant in expression cluster 6 in 12SD80 (36%) and cluster 5 in 12NC29 (20%), both of which showed slightly elevated expression in germinated spores but also significant expression under *in planta* conditions (Table 4; Fig. 4A and B), suggesting that these enzymes have roles throughout development. Interestingly, the clusters with the strongest expression in germinated spores compared to their expression under other conditions (cluster 3 in 12SD80 and clusters 4 and 9 in 12NC29) have relatively low proportions of CAZymes and the highest percentage of predicted apoplast-localized proteins. This may indicate that *P. coronata* f. sp. *avenae* employs a repertoire of apoplastic effectors that do not have enzymatic functions similar to those of CAZymes.

10.1128/mBio.01650-17.4 FIG S4  GO enrichment analysis of secreted proteins. Shown are numbers of genes in enriched GO term classes in the secreted protein sets of 12SD80 and 12NC29. Dot sizes represent the ratio of a given term out of all enriched GO terms, and colors indicate the adjusted *P* value according to the scale insets. Download FIG S4, EPS file, 1.3 MB. Copyright © 2018 Miller et al. 2018 Miller et al. This content is distributed under the terms of the Creative Commons Attribution 4.0 International license.

Glycoside hydrolase (GH) enzymes are a subclass of CAZymes, with 175 and 182 members detected in 12SD80 and 12NC29, respectively (Fig. 3B). Of these, 43 and 46 were predicted to be secreted in 12SD80 and 12NC29, respectively, representing approximately 60% of all secreted CAZymes. The GH5 (cellulase and other diverse enzymatic functions are in this family) and GH47 (α-mannosidases) families were abundant in *P. coronata* f. sp. *avenae*, as seen in *P. graminis* f. sp. *tritici* and *Melampsora larici-populina* (17), with 32 GH5 family members in both isolates and 13 and 18 GH47 family members in 12SD80 and 12NC29, respectively. However, only 2 to 4 members of these families were predicted as secreted, suggesting that these families have mostly intracellular roles. The cellulose-binding module 1 (CBM1) subfamily was not found in *P. coronata* f. sp. *avenae*, consistent with previous observations of rust fungi (17).

Secreted subtilases (serine proteases) and aspartic proteases are predicted to act as effectors in rust fungi and may interfere with plant defense responses (53, 54). Both the A01A (aspartic proteases) and S08A (subtilisin-like serine proteases) families were expanded in the *P. coronata* f. sp. *avenae* genomes, as was found for *P. graminis* f. sp. *tritici* and *Melampsora larici-populina* (17) (26 and 34 members of A01A and 25 and 18 members of S08A in 12SD80 and 12NC29, respectively) (Fig. 3C). A total of 11 (42%) and 17 (50%) aspartic proteases and 17 (68%) and 15 (83%) serine proteases are predicted to be secreted in 12SD80 and 12NC29, respectively. Unlike secreted CAZymes, these secreted proteases have no obvious clustering pattern among differentially expressed secretome genes.

### Variation in effector candidates.

As with genome-wide patterns, heterozygous small variants were more abundant in 1,000-bp upstream and downstream regions than in transcribed regions of effector candidate genes (Fig. S2E). The rate of heterozygous variants was slightly higher for effectors on primary contigs than for all genes on primary contigs in 12NC29, but not in 12SD80, as was the nonsynonymous variant rate (Table 5). Elevated variation rates for effector genes relative to those for all genes were also observed in between-isolate comparisons. SV impacted 13 and 23 predicted effectors on primary contigs in 12SD80 and 12NC29, respectively (Fig. S2F), also with regard to their presence/absence and copy number variation.

**TABLE 5:** Variation rates for annotated genes and predicted effectors on primary contigs in *P. coronata* f. sp. *avenae*

Gene or effector type	No. of variants/kbp for indicated isolate	
	12SD80	12NC29
Heterozygous variants for all genes	2.83	3.76
Heterozygous variants for effectors	2.86	4.55
Non-synonymous heterozygous variants for all genes	0.98	1.26
Non-synonymous heterozygous variants for effectors	0.93	1.57
Inter-isolate variants for all genes	6.37	5.01
Inter-isolate variants for all effectors	7.39	5.88
Inter-isolate variants for orthologous genes	6.20	4.95
Inter-isolate variants for orthologous effectors	7.76	5.86

Orthologous gene relationships for effectors were identified to examine the conservation of effector repertoires between haplotypes and isolates. Approximately 50% of predicted effectors had an allele pair (Table 3; see Dataset S1 to S4 at https://github.com/figueroalab/Pca-genome), while a total of 91 (11%) and 123 (14%) predicted effectors were haplotype singletons in 12SD80 and 12NC29, respectively (Table 3; see Dataset S5 to S8 at https://github.com/figueroalab/Pca-genome). For 12SD80, 336 predicted effector genes on primary contigs had orthologs in 12NC29 (primary contigs and haplotigs), while 184 were isolate singletons, and similar numbers were observed for the reciprocal comparison (Table 3; see Dataset S9 to S12 at https://github.com/figueroalab/Pca-genome). Inter-isolate variation rates for orthologous effector genes were slightly elevated compared to those for all orthologous genes (Table 5). Overall, these results showed substantial variation in effector gene candidates between both haplotypes and isolates, which may provide a basis for virulence differences between the isolates.

### Conservation of expression patterns between secreted orthologous proteins.

When the secretome expression clusters for each isolate were overlaid with orthology relationships, the majority of orthologous secreted proteins and predicted effectors showed conserved expression patterns between 12SD80 and 12NC29 (Fig. 4C to F; Fig. S5 and S6). For instance, orthologs of genes in cluster 4 of 12SD80 with the strongest haustorial expression relative to that of germinated spores were found mainly in cluster 3 in 12NC29, which showed the same expression profile (Fig. 4C). A number of orthologs were also found in 12NC29 cluster 6, which shows the next strongest haustorial expression, while there was a single ortholog in 12NC29 cluster 1 that was slightly upregulated in haustoria compared to its expression under all other conditions. Similar conservation of expression profiles were observed for 12NC29 genes in cluster 3, which showed strong conservation of expression patterns to 12SD80 clusters 4 and 5 (Fig. 4D). Genes in 12SD80 cluster 5 (the second strongest haustorial cluster) showed orthology mostly to genes in the equivalent cluster 6 in 12NC29, although some orthologs were in clusters 1 and 3 (Fig. 4E). For 12NC29 cluster 6, expression tended to be conserved with that of 12SD80 cluster 5 (Fig. 4F). A few orthologous effector candidates showed divergent expression patterns between isolates. For instance, one effector in 12SD80 cluster 5 had an ortholog in 12NC29 cluster 4, which has the highest expression in germinated spores, and another had an ortholog in cluster 2 showing highest expression at 5 dpi (Fig. 4E). Such expression differences may contribute to differences in virulence phenotypes. Thus, future investigation of differential expression of orthologous effectors, as well as isolate singleton effectors, may provide key insights into the mechanisms for virulence in *P. coronata* f. sp. *avenae*.

10.1128/mBio.01650-17.5 FIG S5  Secretome clustering and orthology between individual 12SD80 clusters and all 12NC29 clusters. The heatmaps show rlog-transformed expression values for germinated spores (GS), isolated haustoria (H), and infected tissues at 2 dpi (2d) and 5 dpi (5 days), with dark blue indicating high expression according to the scale inset. Links depict orthology relationships between secretome genes (gray lines) and effectors (red lines) in all 12NC29 clusters and 12SD80 clusters 1 (A), 2 (B), 3 (C), 6 (D), and 7 (E). Download FIG S5, PDF file, 1.1 MB. Copyright © 2018 Miller et al. 2018 Miller et al. This content is distributed under the terms of the Creative Commons Attribution 4.0 International license.

10.1128/mBio.01650-17.6 FIG S6  Secretome clustering and orthology between individual 12NC29 clusters and all 12SD80 clusters. The heatmaps show rlog-transformed expression values for germinated spores (GS), isolated haustoria (H), and infected tissues at 2 dpi (2d) and 5 dpi (5 days), with dark blue indicating high expression according to the scale inset. Links depict orthologous relationships between secretome genes (black lines) and effectors (red lines) in all 12SD80 clusters and 12NC29 clusters 1 (A), 2 (B), 3 (C), 6 (D), and 7 (E). Download FIG S6, PDF file, 1.1 MB. Copyright © 2018 Miller et al. 2018 Miller et al. This content is distributed under the terms of the Creative Commons Attribution 4.0 International license.

### Genomic context of predicted effector candidate genes.

**FIG 5:**
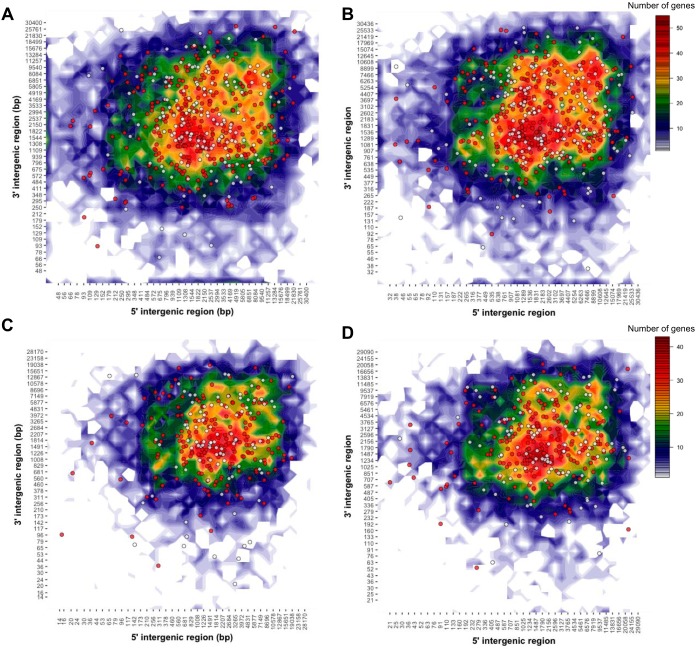
Genomic landscape of predicted *P. coronata* f. sp. *avenae* effectors. Heatmap plots representing the distribution of 5′ and 3′ intergenic distances for all genes on primary contigs of 12SD80 (A) and 12NC29 (B) and haplotigs of 12SD80 (C) and 12NC29 (D). Scales representing gene content per bin are shown on the right. Circles indicate predicted effectors with orthologs (red) or isolate singletons (white).

Genome sequences of several filamentous plant pathogens have provided evidence for a “two-speed genome” model in which rapidly evolving effector genes are preferentially located in low-gene-density and repeat-rich regions (55). This genome architecture may favor fast host adaptation by relieving constraints on effector diversification. To determine the distribution of genes in gene-rich or -sparse regions, we used a two-dimensional genome-binning method (56) to plot intergenic distances for all genes in *P. coronata* f. sp. *avenae* (Fig. 5). Predicted effectors on primary contigs and haplotigs in both isolates showed no difference in location from locations in the overall gene space. Moreover, both orthologous effector genes and isolate singletons had intergenic distances similar to those of all genes. Genome-wide geometric correlation with the GenometriCorr R package (57) found no significant association between effector genes and repeat elements in either isolate. Thus, these findings do not support the presence of a “two-speed genome” in *P. coronata* f. sp. *avenae*, consistent with observations for other rust fungi (58).

### Conclusions and future directions.

A significant challenge when assembling dikaryotic fungal genomes is to capture and align haplotype variation. Here, we demonstrate successful implementation of the diploid-aware long-read assembler FALCON and FALCON-Unzip to generate highly contiguous genome assemblies and resolve haplotypes from SMRT sequencing data for the oat crown rust fungus, *P. coronata* f. sp. *avenae*. These phased assemblies allowed detection of structural variation between haplotypes equivalent to more than 2% of the genome size, which impacted a significant number of genes and predicted effectors. This type of variation has not been previously examined in rust species due to the limitations imposed by collapsed short-read genome assemblies. Furthermore, the long-read assembly approach greatly improved contiguity compared to that of short-read assemblies of other rust fungi, which are highly fragmented due to an abundance of repetitive sequences in their genomes. Orthology analysis also allowed detection of allele pairs on the different haplotypes, as well as many genes potentially unique to one haplotype or highly diverged. We also observed high divergence in gene content and sequence between isolates, which may reflect their origins from geographically separated populations (South Dakota versus North Carolina). Transcriptome profiling revealed clusters of haustorially expressed secreted proteins that are likely enriched for host-delivered effectors, as well as clusters of predicted CAZymes and apoplastic effectors that are preferentially expressed in germinated urediniospores.

Several mechanisms, including mutation, sexual recombination, and somatic hybridization are postulated to cause changes in virulence phenotypes in rust fungal populations (14, 16). However, few studies have specifically characterized molecular events associated with virulence variation, and large-scale, whole-genome comparative population analyses have not been conducted for rust fungi. The high-quality, haplotype-phased genome references for two dikaryotic *P. coronata* f. sp. *avenae* isolates developed in this study provide the foundation for large-scale resequencing of *P. coronata* f. sp. *avenae* isolates to identify genetic variation underlying variability in virulence phenotypes. The identification of the *Avr* genes corresponding to known oat *R* genes will help to prioritize and pyramid broadly effective *R* genes in oat-breeding programs.

## MATERIALS AND METHODS

### *Puccinia coronata* f. sp. *avenae* isolates and plant inoculations.

*P. coronata* f. sp. *avenae* isolates 12NC29 (pathotype LBBB) and 12SD80 (pathotype STTG) were collected from North Carolina and South Dakota, respectively, by the USDA-ARS Cereal Disease Laboratory (CDL) annual rust surveys in 2012 and stored at −80°C. To ensure isolate purity, two single-pustule purifications from low-density infections on 7-day-old oat seedlings (variety Marvelous) were completed prior to amplification of urediniospores as described by Carson (6). Heat shock-activated (45°C, 15 min) urediniospores were resuspended in Isopar M oil (ExxonMobil) at 2 mg spores/ml for spray inoculation (50 µl per plant). Inoculated plants were placed in dew chambers in the dark overnight (16 h) with 2 min of misting every 30 min and then maintained in isolated growth chambers (18 h/6 h of light/dark, 22/18°C for day/night, 50% relative humidity). Pathotype assignment and final assessments of the identity and purity of each isolate was performed using standard oat differential lines (2, 7), with infection types of “0,” “0;,” “;,” “;C,” “1;,” “1,” “2,” “3,” “3+,” and “4” converted to a 0-to-9 numeric scale, respectively, for heat map generation.

### DNA extraction from *P. coronata* f. sp. *avenae* urediniospores for Illumina and PacBio sequencing.

Freshly harvested urediniospores were germinated as described previously (59), and fungal mats were vacuum dried, lyophilized, and stored at −80°C. The lyophilized tissue was ground in liquid nitrogen in 20- to 30-mg batches in 2-ml microcentrifuge tubes. DNA was extracted using genomic-tip 20/G columns (Qiagen catalog number 10223) by following a user-supplied protocol (https://www.qiagen.com/us/resources/resourcedetail?id=cb2ac658-8d66-43f0-968e-7bb0ea2c402a&lang=en), except that lysis buffer contained 0.5 mg/ml of lysing enzymes from *Trichoderma harzianum* (Sigma; L1412) and DNA was resuspended in Qiagen EB. Qubit (Invitrogen) and pulsed-field gel electrophoresis with a CHEF-DR III apparatus (Bio-Rad) were used to evaluate DNA quantity and quality, with yields of 15 to 20 µg per 200 mg of tissue obtained.

### Genomic DNA sequencing and *de novo* assembly.

Approximately 10 µg of genomic DNA was purified with AMPure XP beads (Beckman Coulter, Inc.) and sheared to an average size of 20 kbp using g-TUBEs (Covaris). Size and quantity were assessed using the 2200 TapeStation instrument (Agilent Technologies). Library preparation was carried out according to the PacBio standard 20-kbp protocol, with size selection performed using a BluePippin sequencer (Sage Science) with a 0.75% agarose cassette and a lower cutoff of 7 kbp. Twenty-five SMRT cells per library were run on the PacBio RSII sequencer (Pacific Biosciences) using P6/C4 chemistry, a 0.15 nM MagBead loading concentration, and 360-min movie lengths at the Frederick National Laboratory for Cancer Research (Frederick, MD, USA). Illumina libraries were prepared from 100 ng of genomic DNA with the TruSeq Nano DNA procedure and a 350-bp insert size. Both libraries were multiplexed and sequenced in one lane (HiSeq 2500, rapid-run mode, 100-bp paired-end reads) at the University of Minnesota Genomics Center (UMGC) using Illumina Real-Time Analysis software version 1.18.64 for quality-scored base calling.

SMRT reads were assembled using FALCON version 0.7.3 (https://github.com/PacificBiosciences/FALCON-integrate/tree/funzip_052016). After several trial assemblies, a set of parameters with a relatively stringent overlap length was selected to reduce misassembly of repetitive regions while maintaining a high contiguity (see https://github.com/figueroalab/Pca-genome for the FALCON config file used for assembly). The settings that we used were based on configuration files recommended by PacBio (in particular, the *Arabidopsis* config file at https://github.com/PacificBiosciences/FALCON/tree/master/examples). However, we increased our overlap size substantially to avoid misassembly of repeats, which are abundant in rust fungal genomes. We assessed three different overlap cutoffs for the pa_HPCdaligner_option (for the “–l” parameter, we used cutoffs of 4,800, 2,400, and 1,000) and then examined contig numbers and *N*_50_ values for each assembly. We settled on the higher cutoff value of 4,800 bp to ensure assembly correctness, although this reduced contiguity, as is reflected in the lower *N*_50_ and higher contig numbers. Read length cutoffs of 9,691 bp for 12NC29 and 8,765 bp for 12SD80 were auto-computed by FALCON based on the seed coverage and expected genome size in the config file. After assembly, FALCON-Unzip (31) was used to phase haplotypes and generate consensus sequences for primary contigs and haplotigs using default parameters. Primary contigs and haplotigs were polished using the Quiver algorithm and corrected for SNPs and indels using Illumina data via Pilon with the parameters “–diploid” and “–fix all” (60).

Low-quality contigs (over 20% of their size was masked by Quiver [less than 100 kbp]) were removed using custom python scripts. To select this cutoff, we carefully analyzed the size distribution of contigs against levels of masking and determined that 20% was an acceptable tradeoff between preserving length and removing poor-quality contigs. This filtering step resulted in the removal of 64 and 58 primary contigs for 12SD80 and 12NC29, respectively. We mapped Illumina sequencing reads to the genomes before and after Quiver filtering and found only 0.5% and 0.09% drops in the mapping rates of 12SD80 and 12NC29, respectively, indicating that very few unique genomic regions were removed during filtering.

Eleven contigs from 12NC29 and two contigs from 12SD80 with significant hits to nonfungal organisms (BLAST search against the NCBI nr/nt database followed by manual inspection of all nonfungal hits) were excluded as contaminants. Final assembly metrics were derived using QUAST version 4.3 (61), and the Integrative Genomics Viewer (IGV) (62) was used to visualize haplotig regions in primary contigs. To evaluate assembly completeness, the fungal lineage set of orthologs in the software BUSCO (v2.0) (35) was used for comparison, with *Ustilago maydis* as the species selected for AUGUSTUS gene prediction.

### RNA isolation.

Seven-day-old oat seedlings were inoculated with 10 mg spores/ml or mock inoculated with oil. Three leaves were pooled per biological replicate at 2 and 5 days postinoculation (dpi), frozen in liquid nitrogen, and kept at −80°C. Haustoria were isolated from infected leaves at 5 dpi (inoculated with 20 mg spores/ml) as previously described (18) and stored at −80°C. Prior to RNA extraction, haustorial cells were resuspended in 500 µl of RLT lysis buffer (Qiagen), transferred to FastPrep lysing beads (MP Biomedicals), and homogenized at 6,000 rpm for 40 s using a bead-beating homogenizer. Germinated urediniospores (16 h) were frozen in liquid nitrogen and kept at −80°C. Three biological replicates were performed for each condition. Samples were ground in liquid nitrogen, and RNA was extracted using the RNeasy plant minikit (Qiagen) according to the manufacturer’s protocols. RNA quality was assessed using an Agilent 2100 Bioanalyzer.

### RNA sequencing and transcriptome assembly.

Strand-specific RNA library construction and sequencing (Illumina HiSeq 2500, 125-bp paired-end reads) were carried out at the UMGC. Libraries from germinated spores, *in planta* infections, and mock conditions were multiplexed across three lanes, while libraries from haustorium samples were multiplexed across two lanes. Short reads and low-quality bases were trimmed using Trimmomatic (63) with the parameters ILLUMINACLIP 2:30:10 LEADING 3, TRAILING 3 SLIDINGWINDOW 4:10, and MINLEN 100. *De novo* transcriptome assembly was performed separately for each isolate by using combined reads from germinated spores, infected plants, and haustoria and Trinity v2.4.0 with the parameters –SS_lib_type RF—normalize_reads (38). The combined reads were also mapped to the assembled genomes of each isolate using HISAT2 v2.0.5 (64) with the following parameters: –rna-strandness RF—no-mixed. Genome-guided assemblies were generated using Trinity with the following parameters: –SS_lib_type RF—genome_guided_max_intron 3000—normalize_reads.

### Genome annotation.

Each *P. coronata* f. sp. *avenae* assembly (primary contigs and haplotigs combined) was annotated with Funannotate (version 0.6.0, https://github.com/nextgenusfs/funannotate) in diploid mode using transcript evidence from HISAT2 RNA-seq alignments, *de novo* Trinity assemblies, genome-guided Trinity assemblies, and EST clusters from the Department of Energy-Joint Genome Institute (DOE-JGI) for the *Pucciniomycotina* group (downloaded 20 February 2017, http://genome.jgi.doe.gov/pucciniomycotina/pucciniomycotina.info.html). The Funannotate pipeline ran as follows: (i) repeats were identified using RepeatModeler (65) and soft masked using RepeatMasker (66), (ii) protein evidence from a UniProtKB/Swiss-Prot-curated database (downloaded on 26 April 2017) was aligned to the genomes using TBLASTN and exonerate (67), (iii) transcript evidence was aligned using GMAP (68), (iv) *ab initio* gene predictors AUGUSTUS v3.2.3 (69) and GeneMark-ET v4.32 (70) were trained using BRAKER1 (71), (v) tRNAs were predicted with tRNAscan-SE (72), (vi) consensus protein coding gene models were predicted using EvidenceModeler (73), and finally (vii) gene models were discarded if they were more than 90% contained within a repeat masked region and/or identified from a BLASTp search of known transposons against the TransposonPSI (74) and Repbase (75) repeat databases. Any fatal errors detected by tbl2asn (https://www.ncbi.nlm.nih.gov/genbank/asndisc/) were fixed. Functional annotation used available databases and tools, including PFAM (76), InterPro (77), UniProtKB (78), Merops (79), CAZymes (80), and a set of transcription factors based on InterProScan domains (81) to assign functional annotations (there is a full list at https://github.com/nextgenusfs/funannotate). Functional annotations for each isolate were compared (compare function) and summary heatmaps prepared from the parsed results using ComplexHeatmap (1.12.0) in R. Gene ontology (GO) terms were compared between isolates using goatools and Fisher’s exact test with false-discovery rate and multiple-test correction (https://github.com/tanghaibao/goatools).

### Alignment of contigs, identification of collapsed and haplotig-associated regions, coverage analysis, telomeres, and GC content analysis.

Primary contigs and haplotigs were aligned pairwise by using NUCmer (82) with default parameters. A customized script was used to determine coordinates for matches between primary contigs and haplotigs by scanning aligned blocks along the primary contigs and chaining the aligned haplotig blocks located within 15 kbp. Haplotig alignment coordinates were used to generate the collapsed region coordinates with the complement method in BEDtools (v2.25). Alignments between primary contigs without haplotigs and all primary contigs were also conducted by using NUCmer with default parameters, and then self-to-self alignments were filtered out. Illumina DNA sequencing reads were mapped to primary contigs and haplotigs with BWA-MEM version 0.7.12, with default parameters. SAM alignment files were sorted and converted to BAM files with SAMtools (v1.3) (83) and to BED format with BEDtools (v2.25) (84). Coverage of collapsed and haplotig regions was calculated using the BEDtools coverage method. Coverage distributions were plotted as density histograms with the ggjoy package in R. The GC content of all contigs was calculated and the distribution plotted with the hist function in R. Telomeres were identified by the presence of at least 10 repeats of CCCTAA or TTAGGG within 200 bp of the end of a contig using a custom script.

### Genome-wide heterozygosity and variant analysis.

Small variants (SNPs and indels) were identified by mapping Illumina DNA sequencing reads to only the primary contigs of each assembly by using BWA-MEM version 0.7.12 with default parameters. PCR duplicates were removed using SAMtools (v1.3) (83), and SNPs were called using FreeBayes (v1.1.0) (85). SNPs were filtered using vcflib (v1.0.0-rc1, https://github.com/vcflib/vcflib) with parameters (QUAL > 20 & QUAL/AO > 10 & SAF > 0 & SAR > 0 & RPR > 1 & RPL > 1 & AB > 0.2 & AB < 0.8) within isolates or (QUAL > 20 & QUAL/AO > 10 & SAF > 0 & SAR > 0 & RPR > 1 & RPL > 1) between isolates. Variants were annotated for genomic location and functional impact using ANNOVAR (16 July 2017 version) (86).

*k*-Mer counts (21 bp) were generated with Jellyfish (v2.1.3) from raw Illumina DNA sequencing data of *P. coronata* f. sp. *avenae* isolates as well as Illumina sequencing data downloaded from the NCBI SRA for the rust species: *Melampsora larici-populina* (SRR4063847) (17), *Puccinia striiformis* f. sp. *tritici* (SRR058505 and SRR058506) (19), *Puccinia triticina* (SRR027504 and SRR027505), and *Melampsora lini* (22). The resulting histograms were used as input for GenomeScope (42).

To identify structural variations (SVs), haplotigs were aligned to primary contigs with MUMmer (v3.23) with the following parameters: nucmer -maxmatch -l 100 -c 500 (82). SVs were detected with Assemblytics (43) by using default parameters with a minimum variant size of 50 bp, a maximum variant size of 10 kbp, and a unique sequence length for anchor filtering of 10 kbp.

### Identification of alleles and orthologs between isolates.

Proteinortho (39) with the parameters -e 1e-05 -synteny -singles was used to identify orthologous groups and singletons based on an all-against-all BLASTp search of all annotated genes in 12SD80 and 12NC29, followed by construction of an edge-weighted directed graph (edge weight is the BLAST bit score) and heuristic identification of maximal complete multipartite subgraphs. Protein nodes included in subgraphs were defined as orthologous groups. Orthologous genes located in homologous haplotig and primary contig regions based on a gene annotation (gff3) file were assigned as allele pairs. Singletons on primary contigs were subjected to a BLAST search against corresponding haplotigs to determine how many were truly absent in one haplotype versus present but highly varied or fragmented. An E value of <0.00001 was used to identify significant BLAST hits.

### Secretome and effector prediction and expression analysis.

Secreted proteins were predicted using a method sensitive to fungal effector discovery (87) based on (i) the presence of a predicted signal peptide using SignalP-NN 3.0 (88), (ii) a TargetP localization prediction of “secreted” or “unknown” (with no restriction on the RC score) (89), and (iii) no transmembrane domain outside the signal peptide region (with TMHMM 2.0) (90). Secreted effectors were predicted using EffectorP 1.0 (45). FeatureCounts (91) was used to generate read counts for each gene from RNA-seq data, and genes differentially expressed in either haustoria or infected leaves relative to germinated spores (|log fold change| > 1.5 and an adjusted *P* value < 0.1) were identified using the DESeq2 R package (92). *k*-Means clustering was performed on average rlog-transformed values for each gene and condition. The optimal number of clusters was defined using the elbow plot method and circular heatmaps drawn using Circos (93). GO enrichment analysis was carried out with the enrichGO function in the R package clusterProfiler version 3.4.4 (94) by the molecular function ontology method and the Holm method to correct *P* values for multiple comparisons. Categories were considered significantly enriched if they had an adjusted *P* value of less than 0.01 and a *q* value of less than 0.05. Functional enrichments of collapsed, allelic, and singleton genes were tested as described above.

Local gene density was assessed by the method of Saunders et al. (56), with updates from density-Mapr (https://github.com/Adamtaranto/density-Mapr) to plot the 5′ and 3′ intergenic distance for each gene. The R package GenometriCorr (57) was used to test for associations between effectors and various categories of repeats within 10-kbp regions using default parameters.

### Data availability.

All raw sequence reads generated and used in this study are available in the NCBI BioProject (PRJNA398546). This sequencing project has been deposited at DDBJ/ENA/GenBank under the accession numbers PGCI00000000 and PGCJ00000000, and these versions are described in this publication. Genome assemblies and annotations are also available for download at the DOE-JGI Mycocosm Portal (http://genome.jgi.doe.gov/PuccoNC29_1 and http://genome.jgi.doe.gov/PuccoSD80_1). Unless specified otherwise, all scripts and files are available at https://github.com/figueroalab/Pca-genome.
