# Supplementary material for: De Novo Assembly and Phasing of Dikaryotic Genomes from Two Isolates of Puccinia coronata f. sp. avenae, the Causal Agent of Oat Crown Rust
Source: mBio. 2018 Feb 20;9(1):e01650-17. doi: 10.1128/mBio.01650-17 (PMC5821079; doi:10.1128/mBio.01650-17)
Supplement: TABLE S1 [file mbo001183748st1.docx]

**Table S1.** PacBio sequencing and raw read metrics

| **Summary Statistics** | **12SD80** | **12NC29** |
| --- | --- | --- |
| Number of SMRT cells | 25 | 25 |
| Average % ZMW Loading for Productivity 0 | 38.65 | 19.33 |
| Average % ZMW Loading for Productivity 1 | 50.50 | 66.86 |
| Average % ZMW Loading for Productivity 2 | 10.85 | 13.82 |
| Number of Filtered Polymerase Reads | 1,897,431 | 2,511,945 |
| Filtered Polymerase Read bp | 21,009,873,411 | 25,956,073,047 |
| N50 Filtered Polymerase Read Length (bp) | 16,917 | 15,070 |
| Mean Filtered Polymerase Read Length (bp) | 11,072 | 10,333 |
| Number of Filtered Subreads | 3,277,578 | 3,993,633 |
| Filtered Subread bp | 20,943,700,115 | 25,884,744,697 |
| Mean Filtered Subread length (bp) | 6,389 | 6,481 |
| N50 Filtered Subread length (bp) | 8,445 | 8,609 |
