# Supplementary figures and images for: De Novo Assembly and Phasing of Dikaryotic Genomes from Two Isolates of Puccinia coronata f. sp. avenae, the Causal Agent of Oat Crown Rust
Source: mBio. 2018 Feb 20;9(1):e01650-17. doi: 10.1128/mBio.01650-17 (PMC5821079; doi:10.1128/mBio.01650-17)

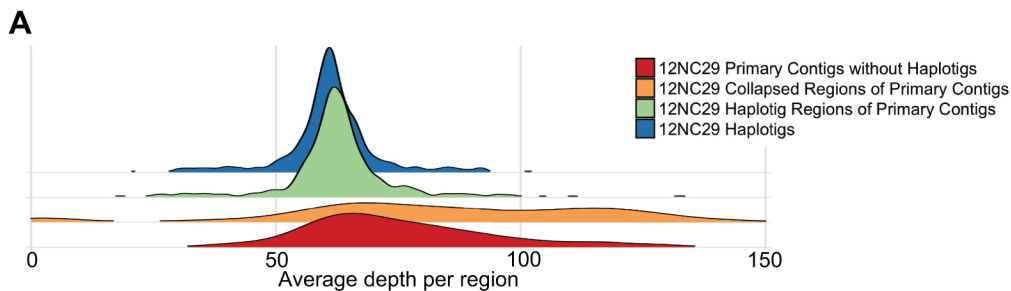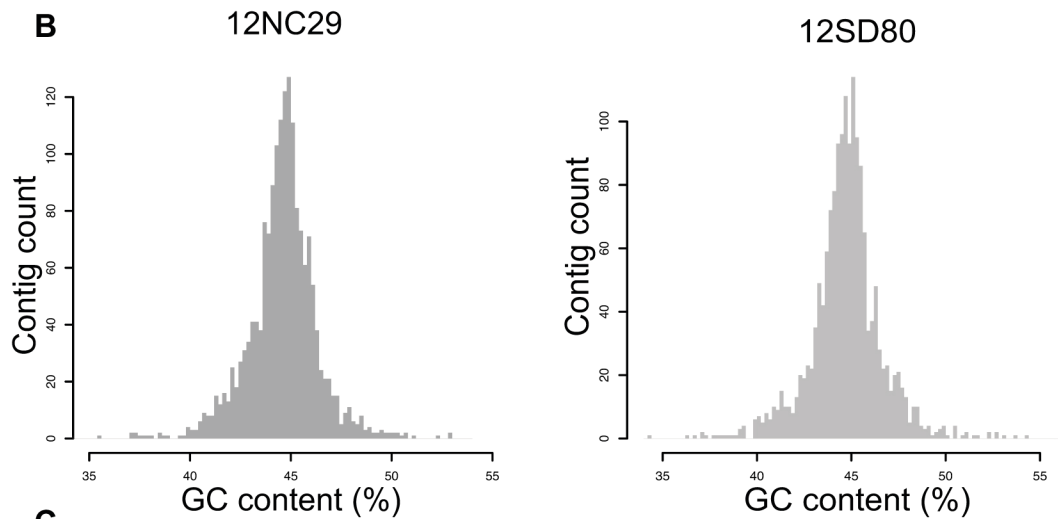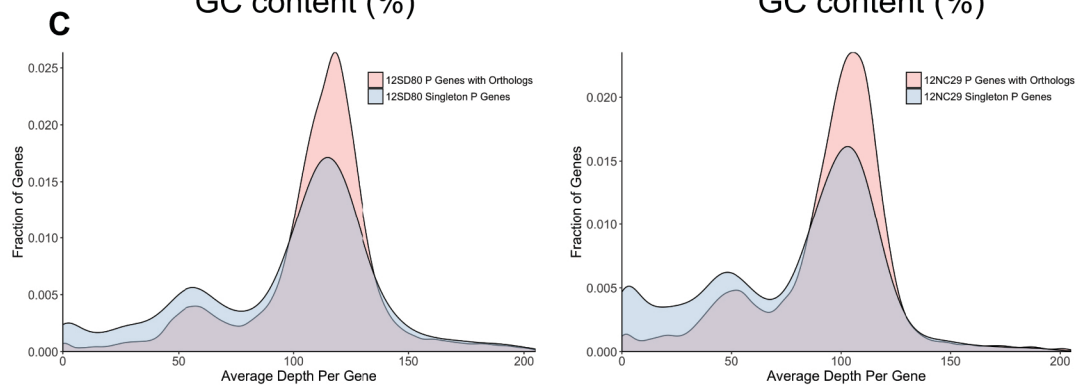

Supplement: FIG S1 [file mbo001183748sf1.pdf]

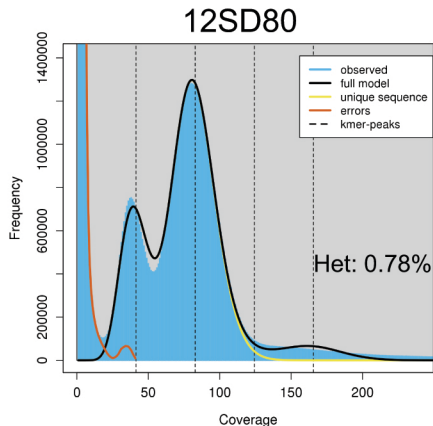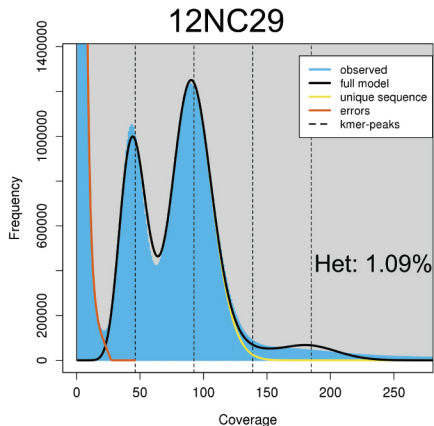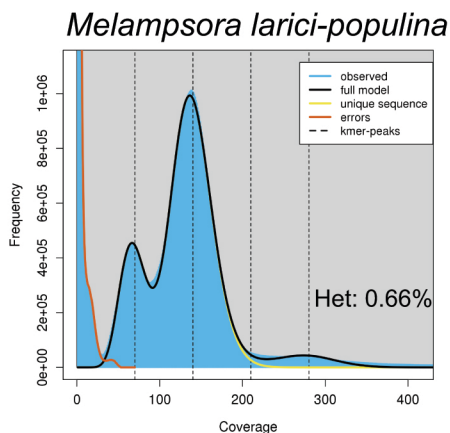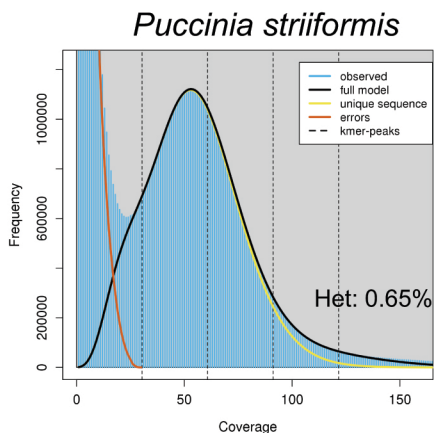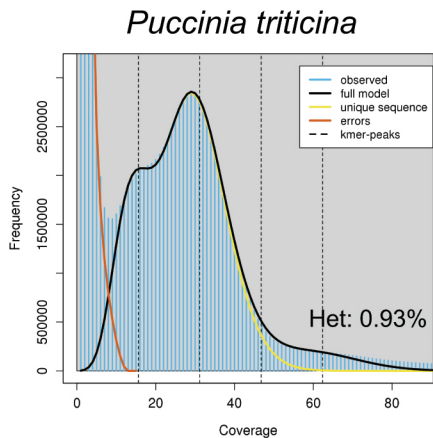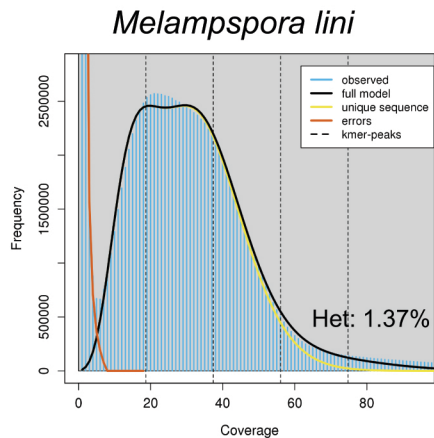

Supplement: FIG S3 [file mbo001183748sf3.pdf]

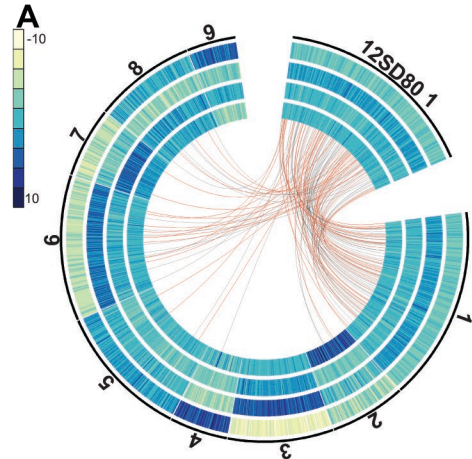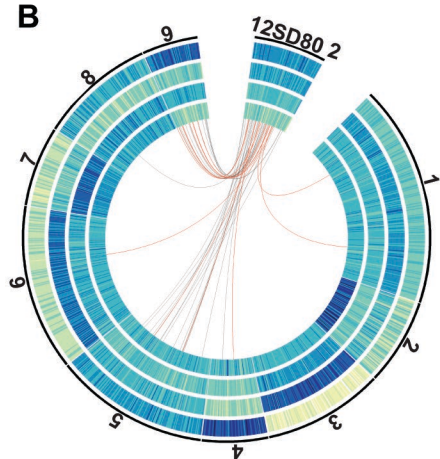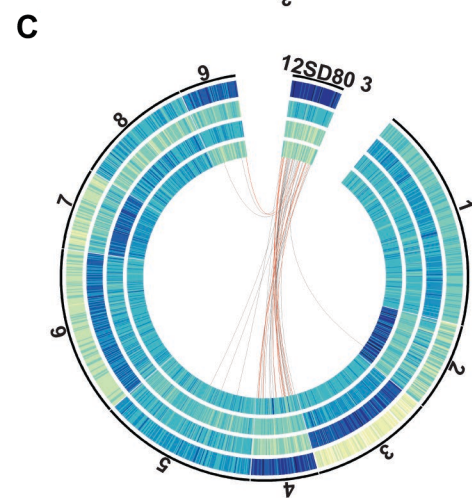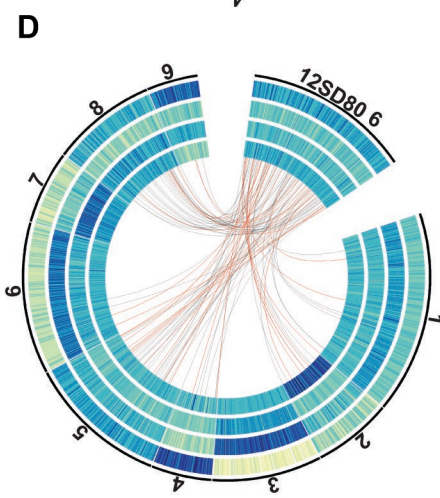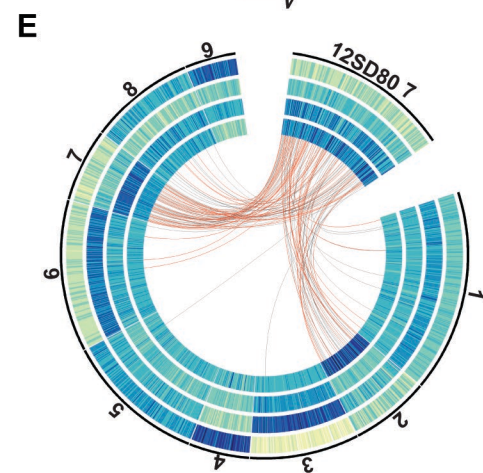

Supplement: FIG S5 [file mbo001183748sf5.pdf]

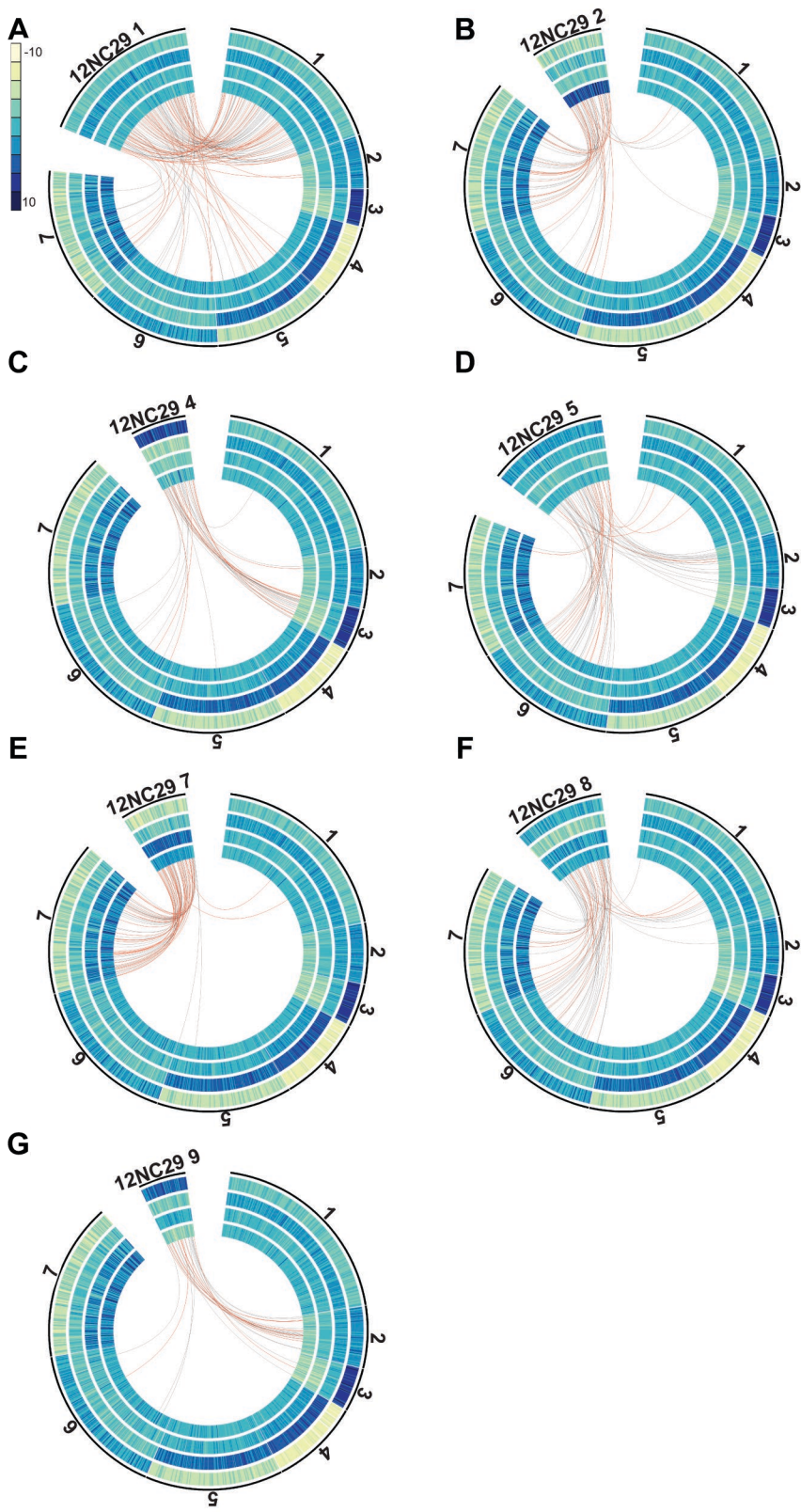

Supplement: FIG S6 [file mbo001183748sf6.pdf]
