# Supplementary material for: De Novo Assembly and Phasing of Dikaryotic Genomes from Two Isolates of Puccinia coronata f. sp. avenae, the Causal Agent of Oat Crown Rust
Source: mBio. 2018 Feb 20;9(1):e01650-17. doi: 10.1128/mBio.01650-17 (PMC5821079; doi:10.1128/mBio.01650-17)
Supplement: TABLE S2 [file mbo001183748st2.docx]

| **Table S2.** Alignment statistics of RNA-seq reads mapping to *Pca* assemblies | | | | | | |  |  |
| --- | --- | --- | --- | --- | --- | --- | --- | --- |
| **Sample** | **Raw reads** | **Clean reads** | **Total mapped reads** | **% Mapped** | | **Mapped unique** | **Mapped multiple** | **Unmapped** |
| *12NC29 libraries mapped to 12NC29 genome assembly (Primary contigs)* | | | | | | | | |
| 12NC29-GS-R1 | 53,172,994 | 50,300,584 | 39,245,696 | | 78 | 31,088,524 | 8,157,172 | 11,054,888 |
| 12NC29-GS-R2 | 51,771,866 | 48,746,084 | 37,785,540 | | 78 | 29,819,852 | 7,965,688 | 10,960,544 |
| 12NC29-GS-R3 | 50,032,216 | 46,686,472 | 35,884,558 | | 77 | 28,416,520 | 7,468,038 | 10,801,914 |
| 12NC29-2-R1 | 52,048,920 | 48,196,376 | 2,787,536 | | 6 | 2,315,308 | 472,228 | 45,408,840 |
| 12NC29-2-R2 | 47,672,740 | 45,506,674 | 2,426,902 | | 5 | 2,008,030 | 418,872 | 43,079,772 |
| 12NC29-2-R3 | 52,943,618 | 50,410,012 | 3,471,570 | | 7 | 2,876,238 | 595,332 | 46,938,442 |
| 12NC29-5-R1 | 48,077,822 | 46,303,514 | 26,803,142 | | 58 | 22,001,928 | 4,801,214 | 19,500,372 |
| 12NC29-5-R2 | 47,777,554 | 45,711,522 | 26,563,974 | | 58 | 21,755,378 | 4,808,596 | 19,147,548 |
| 12NC29-5-R3 | 47,481,362 | 45,087,970 | 26,073,772 | | 58 | 21,402,494 | 4,671,278 | 19,014,198 |
| 12NC29-H-R1 | 162,620,004 | 160,761,346 | 100,875,552 | | 63 | 75,814,094 | 25,061,458 | 59,885,794 |
| 12NC29-H-R2 | 174,113,930 | 172,006,372 | 135,440,370 | | 79 | 97,646,712 | 37,793,658 | 36,566,002 |
| 12NC29-H-R3 | 164,020,012 | 161,993,622 | 125,721,750 | | 78 | 95,095,160 | 30,626,590 | 36,271,872 |
| *12SD80 libraries mapped to 12SD80 genome assembly (Primary contigs)* | | | | | | | | |
| 12SD80-GS-R1 | 28,439,696 | 26,889,866 | 24,271,832 | | 90 | 19,237,220 | 5,034,612 | 2,618,034 |
| 12SD80-GS-R2 | 30,594,492 | 28,994,612 | 26,260,080 | | 91 | 20,850,726 | 5,409,354 | 2,734,532 |
| 12SD80-GS-R3 | 39,270,832 | 36,918,786 | 33,156,814 | | 90 | 26,235,682 | 6,921,132 | 3,761,972 |
| 12SD80-2-R1 | 53,058,730 | 50,519,374 | 859,584 | | 2 | 736,198 | 123,386 | 49,659,790 |
| 12SD80-2-R2 | 55,427,252 | 52,263,028 | 1,682,596 | | 3 | 1,442,138 | 240,458 | 50,580,432 |
| 12SD80-2-R3 | 55,504,734 | 52,161,036 | 1,357,850 | | 3 | 1,165,698 | 192,152 | 50,803,186 |
| 12SD80-5-R1 | 55,261,610 | 52,063,608 | 29,511,114 | | 57 | 25,348,230 | 4,162,884 | 22,552,494 |
| 12SD80-5-R2 | 69,654,590 | 64,547,218 | 35,392,720 | | 55 | 30,326,066 | 5,066,654 | 29,154,498 |
| 12SD80-5-R3 | 51,628,816 | 48,776,396 | 29,283,514 | | 60 | 25,183,762 | 4,099,752 | 19,492,882 |
| 12SD80-H-R1 | 155,330,034 | 150,583,008 | 96,428,996 | | 64 | 56,266,444 | 40,162,552 | 54,154,012 |
| 12SD80-H-R2 | 140,209,738 | 136,167,456 | 49,443,042 | | 36 | 39,990,690 | 9,452,352 | 86,724,414 |
| 12SD80-H-R3 | 150,413,450 | 145,926,896 | 91,078,386 | | 62 | 49,663,028 | 41,415,358 | 54,848,510 |

GS, 2, 5, and H indicate germinated spores, 2 dpi, 5 dpi, and haustoria samples, respectively. R1, R2, and R3 designate the different biological replicates.
