# Supplementary material for: De Novo Assembly and Phasing of Dikaryotic Genomes from Two Isolates of Puccinia coronata f. sp. avenae, the Causal Agent of Oat Crown Rust
Source: mBio. 2018 Feb 20;9(1):e01650-17. doi: 10.1128/mBio.01650-17 (PMC5821079; doi:10.1128/mBio.01650-17)
Supplement: TABLE S3 [file mbo001183748st3.docx]

**Table S3**. GO terms present in predicted effectors on primary contigs.

Redundant GO terms are not displayed.

| **GO Term** | **Description** | **12NC29 Count** | **12SD80 Count** |
| --- | --- | --- | --- |
| GO:0043169 | cation binding | 8 | 4 |
| GO:0008270 | zinc ion binding | 6 | 2 |
| GO:0016853 | isomerase activity | 2 | 2 |
| GO:0008233 | peptidase activity | 1 | 2 |
| GO:0016740 | transferase activity | 1 | N/A |
| GO:0015923 | mannosidase activity | 1 | N/A |
| GO:0046983 | protein dimerization activity | 1 | N/A |
| GO:0000166 | nucleotide binding | 2 | N/A |
| GO:0003735 | structural constituent of ribosome | N/A | 1 |
